# Supplementary material for: Template-Free Preparation of a Mesopore-Rich Hierarchically Porous Carbon Monolith from a Thermally Rearrangeable Polyurea Network
Source: Int J Mol Sci. 2022 Apr 12;23(8):4271. doi: 10.3390/ijms23084271 (PMC9024688; doi:10.3390/ijms23084271)
Supplement: Supplementary file 1 [file ijms-23-04271-s001.zip › ijms-1663132-supplementary.pdf]

Supplementary Information

Template-Free Preparation of a Mesopore-Rich Hierarchically Porous Carbon Monolith from a Thermally Rearrangeable Polyurea Network

Junsik Nam, Yusin Pak, Gun Young Jung, and Ji-Woong Park*

School of Materials Science and Engineering, Gwangju Institute of Science and Technology, 123 Cheomdangwagi-ro, Buk-gu, Gwangju 61005, Korea

Corresponding author: Ji-Woong Park
School of Materials Science and Engineering, Gwangju Institute of Science and Technology, 123 Cheomdangwagi-ro, Buk-gu, Gwangju 61005, Korea
Telephone: +82-62-715-2315
Fax: +82-62-715-2304
E-mail: jiwoong@gist.ac.kr.

Experimental

Synthesis of rearranged polyurea network (RUN) prepared at various thermal treatment temperatures

As prepared polyurea network (UN) was rearranged with a programmable muffle furnace (Daihan FX-27, Daihan Scientific, Wonju, Korea) at 300, 320, 340, and 360 °C under nitrogen atmosphere, yielding the RUN. The rearranging time of each target temperature was varied with 1, 12, and 24 h, respectively. The temperature increasing rate is 2 °C min^{-1} and the nitrogen gas purging condition is 500 mL min^{-1} for all rearrangement procedures.

Optimization of the carbonization temperature

The pyrolysis temperature of RUN340 was varied among 600, 700, 800, and 900 °C to find optimum pyrolysis temperature for the high electrical conductivity and high specific surface area. The samples were heated to target temperatures with a heating rate of 2 °C min^{-1} and kept for 1 h under the nitrogen atmosphere.

Scanning electron microscope (SEM)

SEM images were collected on SU 70 (Hitachi, Tokyo, Japan). Before taking SEM measurements, samples were dried under a vacuum for 24 h and coated with platinum for 60 seconds using the ion sputter (E-1030, Hitachi, Tokyo, Japan).

Transmission electron microscope (TEM)

TEM was carried out using a model Tecnai F20 ST (FEI, Hillsboro, USA). The TEM samples were prepared with the focused ion beam (FIB) method using Versa 3D DualBeam (FEI, Hillsboro, USA). The samples used for TEM analysis were dispersed in ethanol 4 mg mL^{-1} concentration and loaded on a holey copper grid. The images were collected at 200 kV.

Thermogravimetric analysis (TGA)

Thermogravimetric analysis (TGA) of the samples was carried out using a TA Instruments 2100 series analyzer in the range of 20 ~ 800 °C at a heating rate of 10 °C/min under nitrogen. All samples were dried at 100 °C for 1 hour before the experiment by using TGA.

X-ray photoelectron spectroscopy (XPS)

The chemical state and composition of the products were evaluated using an X-ray photoelectron spectrometer (ESCALAB 250XI, Thermo Fisher Scientific, Waltham, USA), equipped with an Al K-Alpha energy source under ultrahigh vacuum condition with scan number of 3, energy step size of 1.0 eV. For sample preparation, the powder samples were packed in a sample holder with a diameter of 1 cm.

Gas adsorption isotherm

Gas adsorption isotherm curves were recorded on an ASAP 2020 volumetric adsorption apparatus (Micromeritics, Norcross, USA). The nitrogen adsorption-desorption isotherm experiment and carbon dioxide adsorption-desorption isotherm experiment were conducted at 77 K and 273 K, respectively. Before analysis, the samples were degassed using a degassing port of the analyzer at 473 K for 12 h. The surface area and pore size distribution were analyzed using ASAP 2020 v3.00 software (Micromeritics, Norcross, USA). The Brunauer-Emmett-Teller (BET) surface area was calculated from the N_2 adsorption data collected at 77 K. The pore distribution was calculated with the nonlocal density functional theory (NLDFT) N_2 77 K carbon cylinder model. Mesopore surface area was calculated with NLDFT incremental surface area data.

Fourier transform infrared (FT-IR) spectroscopy

FT-IR spectrum was collected on a Nicolet iS10 (Thermo Fisher Scientific, Waltham, USA). The FT-IR samples were prepared in KBr pellets. The KBr pellet was prepared by mixing 1 mg of sample in 100 mg of KBr powder. FT-IR spectra were recorded between 4000 and 1000 cm^{-1}. Each spectrum was obtained by averaging 32 scans recorded at a resolution of 2 cm^{-1}.

Electrode deposition on carbonized RUN (CRUN) monolith

A gold electrode was deposited onto a CRUN monolith via a sputtering process (RF & DC Magnetron Sputtering System, KVS-2003L, Gimpo-si, Korea). The thickness of the electrode was adjusted to 50 nm. The gap between the electrode was 100 μm.

Gas sensing test

Connect the tip to the electrode on the CRUN monolith and seal. Multiple CO_2 adsorption-desorption experiments were conducted by repeating the following experimental procedure. Nitrogen gas was purged by opening the No.1 MFC, the flow rate was 20 mL min^{-1}. 250 seconds after the current was saturated, the No.2 MFC was opened for CO_2 purge. The flow rate of No.1 and No.2 MFC was adjusted to 10 mL min^{-1}. CO_2 was purged until after 250 seconds of saturation. To desorb the CO_2 gas, pure nitrogen gas was purged by closing the No.2 MFC. The No.1 MFC was adjusted to 20 mL min^{-1} for constant gas flow.

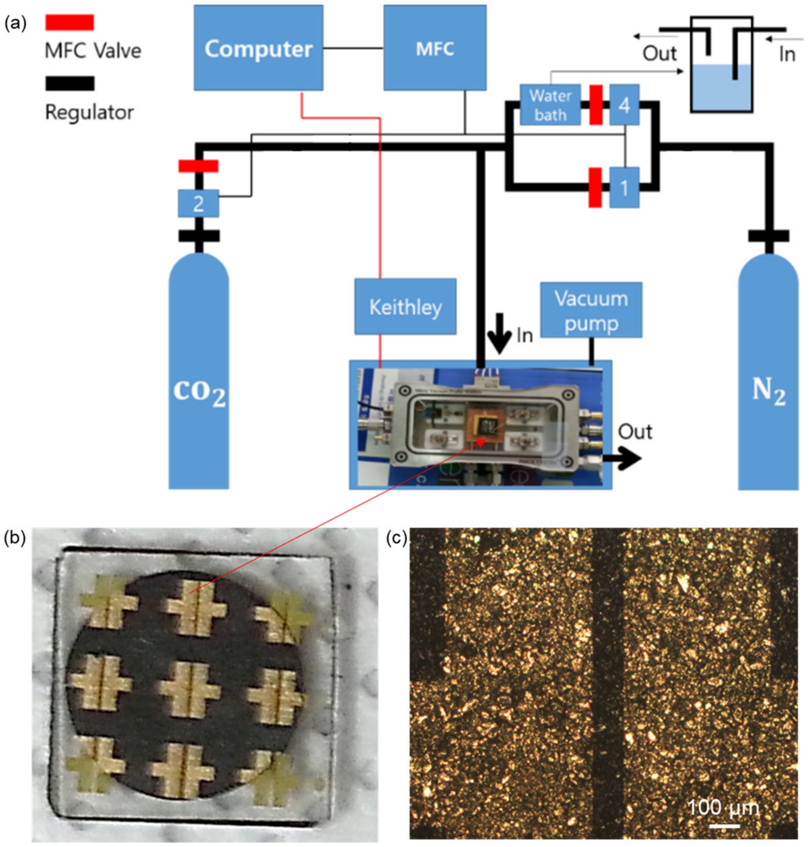

Figure S1. Schematic illustration of gas sensing setup. (a) The setup is divided into four parts; (1) resource: gas and regulators, (2) injector: mass flow controller (MFC), readout system, gas mixer, (3) chamber, and (4) analyzer: Keithley 2400 and homemade Lab-VIEW resistance analyzing program in the computer. (b) Optical image of gold electrode deposited CRUN monolith. (c) Magnified optical microscope image of the electrode deposited CRUN monolith.

Thermogravimetric analysis curve of UN

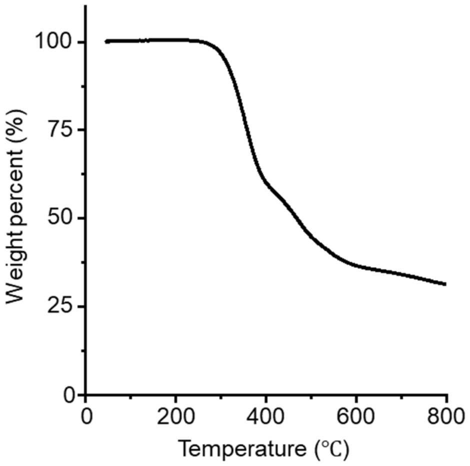

Figure S2. TGA curve of UN.

XPS N1s spectrum of 1-hour treated RUNs at different temperatures

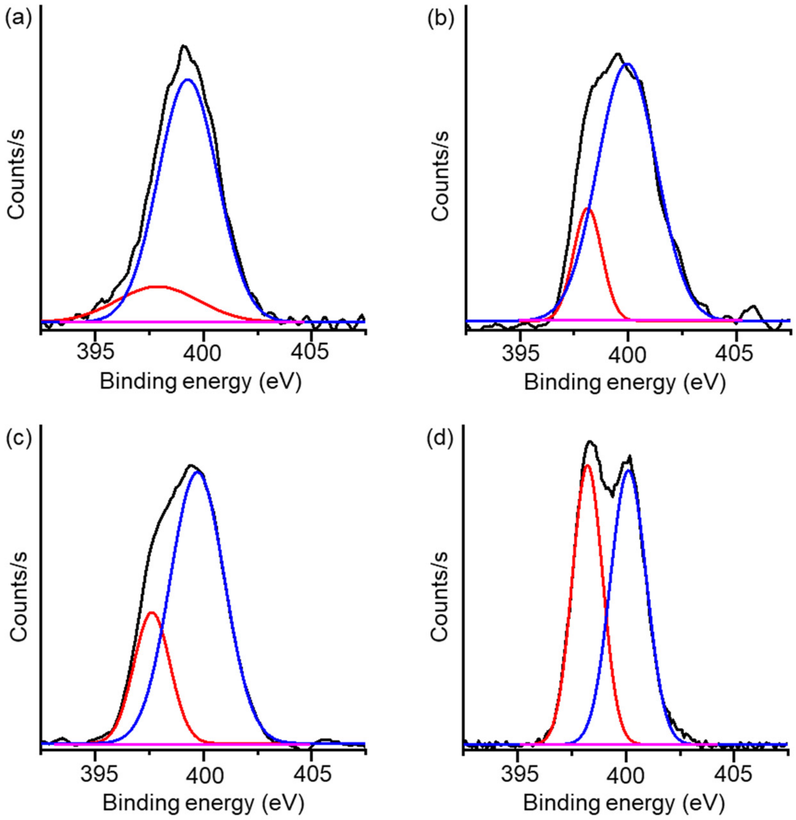

Figure S3. XPS N1s spectrum of 1-hour treated RUNs. (a) RUN treated at 300 °C, (b) RUN treated at 320 °C, (c) RUN treated at 340 °C, and (d) RUN treated at 360 °C. The spectrum was collected with the powder samples, packed in a 1 cm diameter sample holder.

XPS N1s spectrum of 12-hour treated RUNs at different temperatures

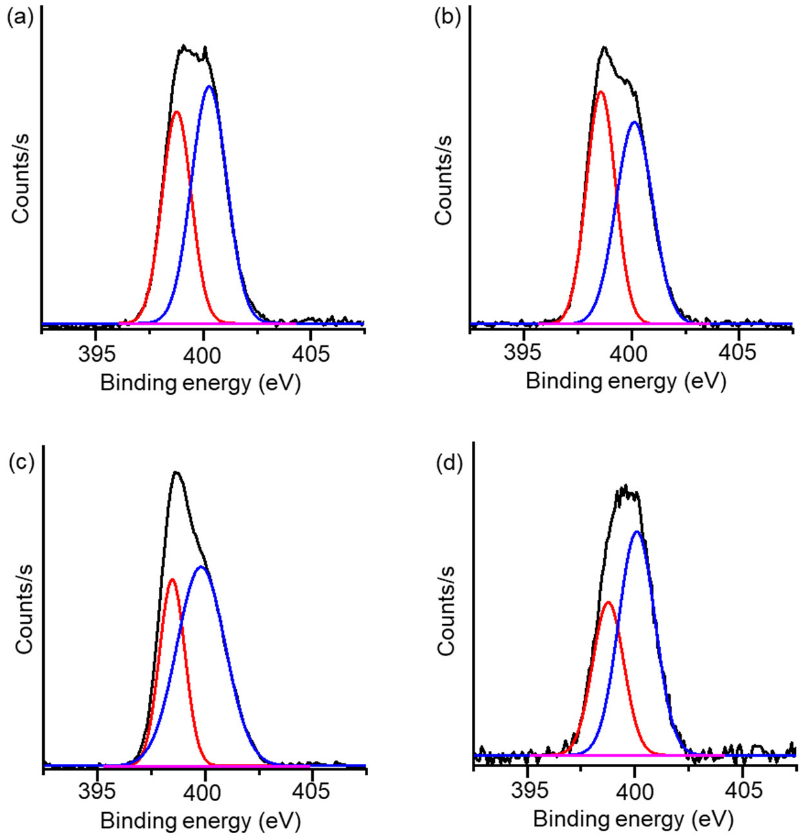

Figure S4. XPS N1s spectrum of 12-hour treated RUNs. (a) RUN treated at 300 °C, (b) RUN treated at 320 °C, (c) RUN treated at 340 °C, and (d) RUN treated at 360 °C. The spectrum was collected with the powder samples, packed in a 1 cm diameter sample holder.

XPS N1s spectrum of 24-hour treated RUNs at different temperatures

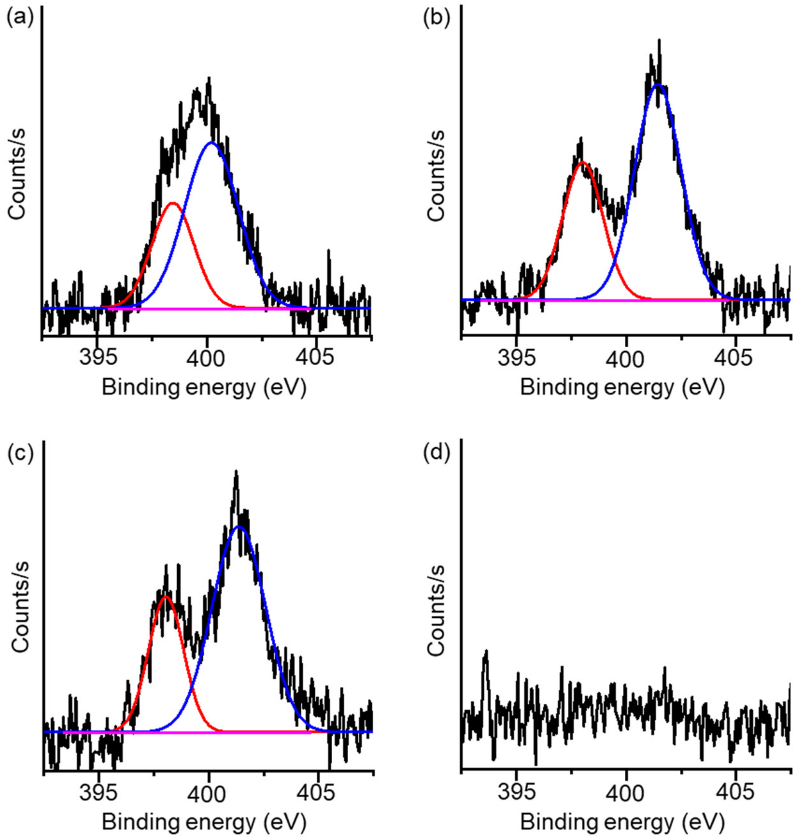

Figure S5. XPS N1s spectrum of 24-hour treated RUNs. (a) RUN treated at 300 °C, (b) RUN treated at 320 °C, (c) RUN treated at 340 °C, and (d) RUN treated at 360 °C. The spectrum was collected with the powder samples, packed in a 1 cm diameter sample holder.

XPS N1s spectra of RUN340 prepared with different durations at the rearranging temperature (340 °C)

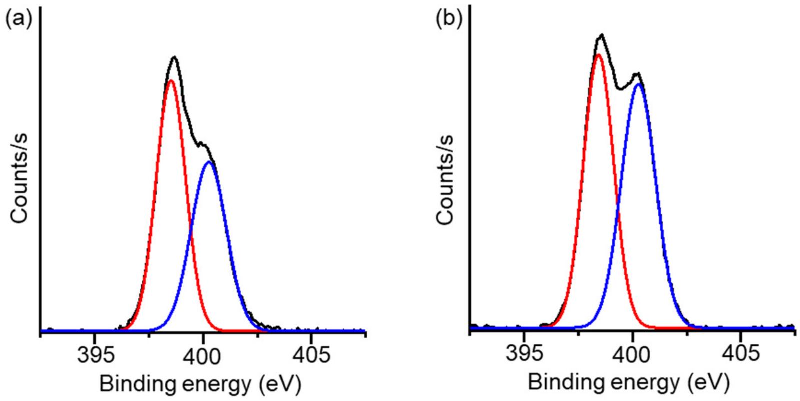

Figure S6. (a) RUN treated at 340 °C for 6 h and (b) RUN treated at 340 °C for 18 h. The spectrum was collected with the powder samples, packed in a 1 cm diameter sample holder.

Porosity and electrical conductivity of the CRUN340 prepared at different carbonization conditions

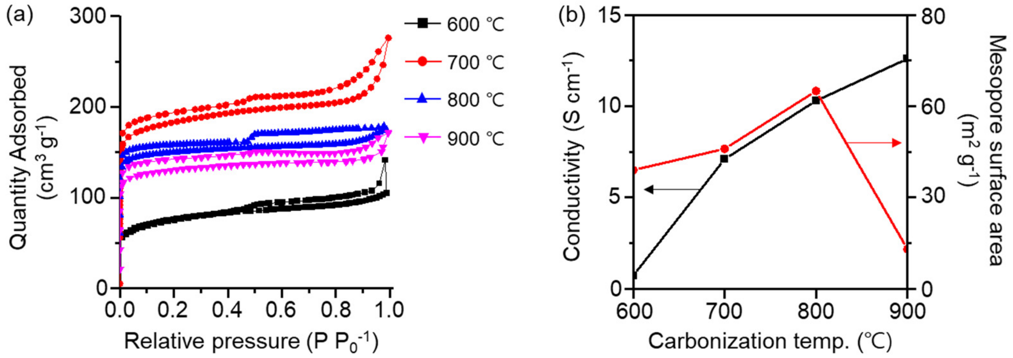

Figure S7. (a) N_2 adsorption-desorption isotherm curves of the CRUN340 prepared at different carbonization temperatures. (b) The electrical conductivity and mesopore surface area of CRUN340s were plotted against the carbonization temperature. The mesopore surface area was calculated with the NLDFT by using the N_2 adsorption isotherm curves.

TEM images of UN

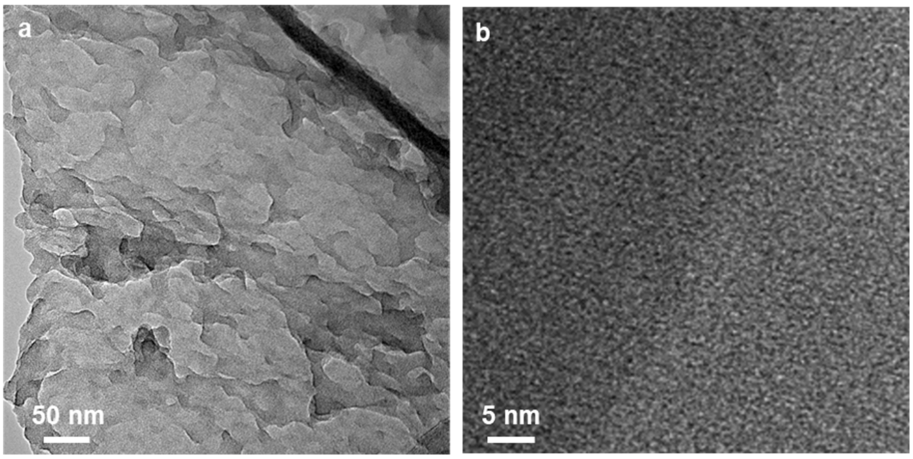

Figure S8. TEM images of a UN monolith. (a) Low magnification TEM image of the UN monolith and (b) high magnification TEM of the UN monolith. The samples used for TEM were prepared with the casting of the UN dispersed ethanol solution on holey copper grids.

Carbon dioxide adsorption amount of CRUNs

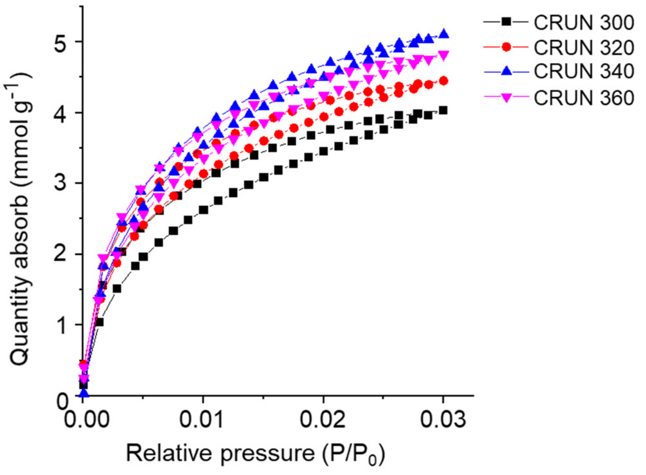

Figure S9. Carbon dioxide adsorption amount of CRUNs.

Table S1. Nitrogen elemental composition and the isocyanurate and urea ratio of the RUNs prepared at different rearrangement temperatures.

Material	Duration at target temp. (hour)	Nitrogen content (wt%)	Isocyanurate (%)	Urea (%)
RUN300	1	13.6	16.8	83.2
	12	12.8	35.7	64.3
	24	3.7	33.2	66.8
RUN320	1	14.1	17.4	82.6
	12	13.1	47.7	52.3
	24	4.4	32.4	67.6
RUN340	1	13.5	24.4	75.6
	6	15.6	51.5	48.5
	12	11.2	55.2	44.8
	18	11.6	43.6	56.4
	24	3.4	29.4	70.6
RUN360	1	10.1	46.5	53.5
	12	6.3	36.5	63.5
	24	0	0	100

*Nitrogen content, isocyanurate, and urea ratio were derived from XPS N1s data.

Table S2. Mesopore surface area, BET surface area, mesopore surface area ratio over BET surface area, micropore volume, and mesopore volume of CRUNs.

Material	S_{meso} (m^2/g)	S_{BET} (m^2/g)	S_{meso}/S_{BET} ratio (%)	V_{micro} (cm^3/g)	V_{meso} (cm^3/g)
CRUN300	20	476	4	0.10	0.038
CRUN320	22	314	7	0.11	0.049
CRUN340	65	600	11	0.24	0.065
CRUN360	21	398	5	0.13	0.018

Table S3. Elemental composition of CRUNs.

**Material**	**Carbon (wt%)**	**Nitrogen (wt%)**	**Oxygen (wt%)**	**Hydrogen (wt%)**
CRUN300	76.32	7.21	14.75	1.71
CRUN320	75.01	7.36	15.71	1.91
CRUN340	75.56	7.58	14.93	1.93
CRUN360	78.09	5.31	14.80	1.79
